# Adipose Mesenchymal Stem Cell‐Derived Exosomes Versus Platelet‐Rich Plasma Treatment for Photoaged Facial Skin: An Investigator‐Blinded, Split‐Face, Non‐Inferiority Trial

**DOI:** 10.1111/jocd.70208

**Published:** 2025-05-25

**Authors:** Blanca Estupiñan, Karen Ly, David J. Goldberg

**Affiliations:** ^1^ Skin Laser & Surgery Specialists, a Division of Schweiger Dermatology Group Hackensack New Jersey USA; ^2^ Department of Dermatology Icahn School of Medicine at Mt. Sinai New York New York USA

**Keywords:** exosomes, platelet‐rich plasma, radiofrequency microneedling

## Abstract

**Background:**

Exosomes, an emerging treatment of interest to aesthetic dermatology, have therapeutic applications in skin rejuvenation, alopecia, atopic dermatitis, acne scarring, and wound healing. Platelet‐rich plasma has been widely utilized for the same indications, among others. Currently, there are no trials comparing the two regenerative modalities in aesthetic dermatology.

**Aims:**

To compare the efficacy and safety of adipose mesenchymal stem cell‐derived (ASC) exosomes versus platelet‐rich plasma for photoaged facial skin.

**Methods:**

An investigator‐blinded, split‐face trial was conducted. Participants with mild to moderate photoaging underwent three radiofrequency microneedling treatments with PRP and topical exosomes each applied to one half of the face.

**Results:**

Both exosomes and PRP equally improved wrinkling, dyschromia, erythema, texture, and overall skin appearance. Histological analysis confirmed increased collagen I and glycosaminoglycans, without significant differences between treatment arms.

**Conclusion:**

ASC exosomes are a promising PRP‐alternative that may be attractive to needle‐averse patients and can hasten the office visit duration, as phlebotomy and centrifugation are not required.

## 1 Introduction

In recent years, regenerative medicine has attracted significant attention due to its potential to harness the body's innate healing mechanisms, particularly for skin rejuvenation [1]. Among the leading biologic therapies under investigation are exosomes and platelet‐rich plasma (PRP), both of which have shown regenerative potential, but their comparative clinical efficacy remains largely unexplored.

Exosomes, a specialized subtype of extracellular vesicles, have demonstrated considerable promise. These nanovesicles are rich in diverse biomolecules, including messenger RNA, microRNA, lipids, metabolites, and proteins, which facilitate intercellular communication and influence recipient cells based on their molecular content. These molecules have been shown to promote collagen synthesis, angiogenesis, and modulation of inflammation, making exosomes a compelling option for rejuvenation of photoaged skin [2].

In dermatology, exosomes have been investigated for their therapeutic applications in skin rejuvenation, alopecia, atopic dermatitis, acne scarring, and wound healing. Studies have also reported enhanced dermal remodeling and improved clinical outcomes when exosomes are combined with microneedling or laser therapy. Additionally, exosomes have shown diagnostic and prognostic utility, underscoring their multifaceted role in advancing dermatological care [3].

Similarly, platelet‐rich plasma (PRP) has been widely studied for its role in tissue healing. PRP is an autologous blood‐derived product containing high concentrations of growth factors, such as PDGF, VEGF, and TGF‐β, cytokines, chemokines, and proteolytic enzymes that are integral to wound healing, promoting fibroblast activation, angiogenesis, and extracellular matrix remodeling [4]. PRP has been extensively studied and is frequently used in combination with modalities such as microneedling and ablative and non‐ablative lasers to enhance skin rejuvenation outcomes. Some clinical trials have demonstrated that PRP, when paired with microneedling, achieves superior results compared to microneedling alone [5, 6, 7]. Similarly, recent studies have shown that exosomes combined with microneedling improve outcomes, further highlighting the potential of these regenerative therapies [8, 9, 10].

Although both treatments are individually promising, few studies have directly compared their efficacy in a controlled clinical setting, and none to date have evaluated their performance in a head‐to‐head, split‐face design with radiofrequency microneedling (RFMN). The split‐face model enables direct comparison within the same subject, minimizing inter‐individual variability. Moreover, RFMN was chosen as the baseline intervention due to its established ability to induce neocollagenesis and dermal remodeling via controlled thermal injury, thereby providing a consistent platform to evaluate the additive effects of either PRP or exosomes [11].

This study is the first clinical trial to evaluate the comparative efficacy of PRP versus exosomes in combination with radiofrequency microneedling for skin rejuvenation. We hypothesize that exosomes are non‐inferior to PRP in improving skin quality, based on their more standardized composition and favorable preclinical results. By exploring these two regenerative modalities, this trial aims to provide critical insights into their respective roles in enhancing skin quality and optimizing aesthetic outcomes.

## 2 Objectives

We conducted an investigator‐blinded, split‐face clinical trial to assess the safety and efficacy of adipose mesenchymal stem cell‐derived (ASC) exosomes compared to PRP after radiofrequency microneedling (RFMN) treatment to improve photoaged facial skin.

## 3 Methods & Materials

### 3.1 Participant Criteria

Fifteen male and female participants aged 18 years or older with a chief concern of photoaging were recruited. Exclusion criteria included participation in another clinical trial, those receiving laser, systemic, or topical prescription treatment of the face within 30 days prior to study treatment initiation. Participants who were pregnant, lactating, or planning to become pregnant were excluded. Participants with known allergies to any components of the study product, untreated skin cancer in the treatment area, dermatitis, or other dermatologic diseases in the treatment area were also excluded. Participants were instructed to discontinue any other skin care products with active ingredients, such as retinol, alpha and beta hydroxy acids, antioxidants, growth factors, or hyaluronic acid. During the study, participants refrained from other cosmetic treatments, tanning bed use, and excessive sun exposure. The study was approved on February 2, 2023, by the Allendale Institutional Review Board, Division of Regulatory and Technical Associates (Old Lyme, CT 06371).

### 3.2 Study Products

The exosome products used in this study were the Exosome Regenerative Complex and the EXO BALM by BENEV (BENEV Company Inc., Mission Viejo, CA). The Exosome Regenerative Complex is created using ExoSCRT technology, which separates and refines 0.1%–0.5% pure exosomes from human adipose stem cells using a two‐step filtration process to remove large particles, cellular debris, proteins, and medium components. To generate the topical solution, one sterile vacuum‐sealed vial of 5 mL diluent is added to a second vial containing 20 mg of lyophilized exosome powder.

EXO BALM is a two‐step post‐treatment system, combining a 20 mg exosome capsule into a post‐treatment cream. The exosome capsule contains human adipose stromal cell exosomes and rose plant stem cell exosomes in addition to growth factors, peptides, amino acids, and humectants. The post‐treatment cream contains additional skin barrier supporting, brightening, and smoothing ingredients including ascorbic acid, tranexamic acid, squalane, hyaluronic acid, and niacinamide, among other ingredients.

For PRP preparation, a platelet‐rich fibrin matrix (PRFM) system was used (Selphyl PRFM, Bethlehem, PA, USA). Venous blood samples were collected via venipuncture in a sterile tube containing acid citrate dextrose anticoagulant. The tube was inverted seven times to ensure complete mixing of blood with the anticoagulant. The tube was then placed in a centrifuge (Drucker centrifuge model #642VFD‐Plus) with appropriate counterbalance and run at 1100 ***g*** for 6 min. The tube was then gently inverted seven times to resuspend platelets into the plasma to generate the PRP. The PRP was then transferred to a second sterile tube containing calcium chloride platelet activator via a Leur Access Device. Once the PRP was completely transferred, the tube was inverted seven times to ensure adequate mixing to generate the PRFM. This product contains at least 2 x the concentration of platelets compared to whole blood samples. This is because the PRFM product filters out RBC/WBCs. As the fibrin matrix is broken down, the platelets are activated and there is a sustained release of growth factors up to 7 days [12].

### 3.3 Design Overview

This was an investigator‐blinded, non‐randomized, split‐face clinical trial conducted at a single outpatient dermatology clinical research center. After obtaining informed consent, participants underwent clinical multiple‐angle standardized photographs at baseline, 3–6 months post‐treatment.

Participants received three full‐face RFMN treatments (SYLFIRM X device, BENEV, Mission Viejo, CA) at 4‐week intervals. Prior to treatment, topical anesthesia with benzocaine 20%/lidocaine 10%/tetracaine 10% cream was applied for 30 min. A split‐face design was used to allow participants to act as their own control in order for precise evaluation and to reduce variability due to age, skin type, and differences in healing. The study treatments were applied carefully without crossing the midline of the face immediately post‐treatment. PRP was applied to the right side of the face and exosome product was applied to the left side of the face. Participants were instructed to use only petroleum jelly on the right side of the face twice daily and 1 pump of a post‐treatment recovery exosome cream twice daily to the left side of the face for 10 days.

### 3.4 Clinical Evaluation

Blinded investigators viewed clinical images and completed questionnaires at each visit. The overall skin appearance, wrinkling, dyschromia, erythema, and textural quality of the skin on each side of the face were graded on a 5‐point scale ranging from 0 (lowest quality/worst appearance) to 5 (highest quality/best appearance). Investigators also graded photodamage using the validated Griffiths Photonumeric Photoaging scale, which features a 9‐point scale ranging from 0 (no photodamage) to 8 (severe atrophic photodamage). Adverse events were assessed and documented as mild, moderate, or severe. Investigators periodically reviewed adverse events to assess if study continuation was appropriate.

### 3.5 Histological Evaluation

The first 10 participants enrolled underwent 3 mm punch biopsies of the right and left infraauricular area at baseline, 3, and 6 months post‐treatment. Specimens were stained with hematoxylin and eosin to evaluate pre‐ and post‐treatment skin. Measurements of type I and type III collagen and glycosaminoglycans (GAGs) were evaluated by a pathologist using collagen I and III and Alcian blue pH 2.5 stains, respectively. Tissues were scored for subjective stain scores as follows: 0 = no stain (background), 1 = minimal, 2 = mild, and 3 = moderate stain intensity.

Automated image analysis was completed with Indica Labs HALO software using the Area Quantification module. In developing the Area Quantification algorithm, the positive stain was selected by the user, and thresholds for strong positive, moderate positive, and weak positive staining were set. The software then collated pixels into intensity bins, which were reported in positive area (microns^2^) and percent positive staining, respectively.

The change between the pre‐treatment group and all other time points was calculated for both pathology scores and image analysis percent positive tissue for each individual. Group averages were calculated for each pathologist score on each stain for the four time points. Mean and standard error for the image analysis quantitation values were calculated for the participants at each time point.

### 3.6 Safety Evaluation

Investigators assessed and documented any clinically significant increases in side effects attributed to the treatments. There was periodic review and determination of whether the overall incidence of all adverse events reported during the study was acceptable.

### 3.7 Statistical Analysis

Given the small sample size, statistical analyses were not performed for investigator clinical assessments. A student's two‐tailed *t*‐test with an α of 0.05 was used to compare the percentages of histological positive tissue staining for collagen I, collagen III, and GAGs between treatment arms.

## 4 Results

Fifteen participants, aged 44–68 years old, enrolled in the trial. One patient discontinued due to withdrawal of consent for punch biopsies. Fitzpatrick skin types ranged from I to IV. The RFMN settings used were as follows: continuous wave (CW) mode, setting 2 (160 ms) or 3 (200 ms), power level 4, depth 0.7–1.5 mm. The same settings were used on both sides of the face for each patient. There were no adverse events. Expected side effects, including transient mild pain, erythema, edema, and light crusting, resolved in less than 1 week.

At baseline, the average investigator ratings of overall skin appearance, wrinkling, dyschromia, erythema, and texture were equivalent on both the right and left sides of the face. The Griffiths' Photonumeric Photoaging Scale scores were the same on both sides of the face.

### 4.1 Investigator Assessments

**FIGURE 1:**
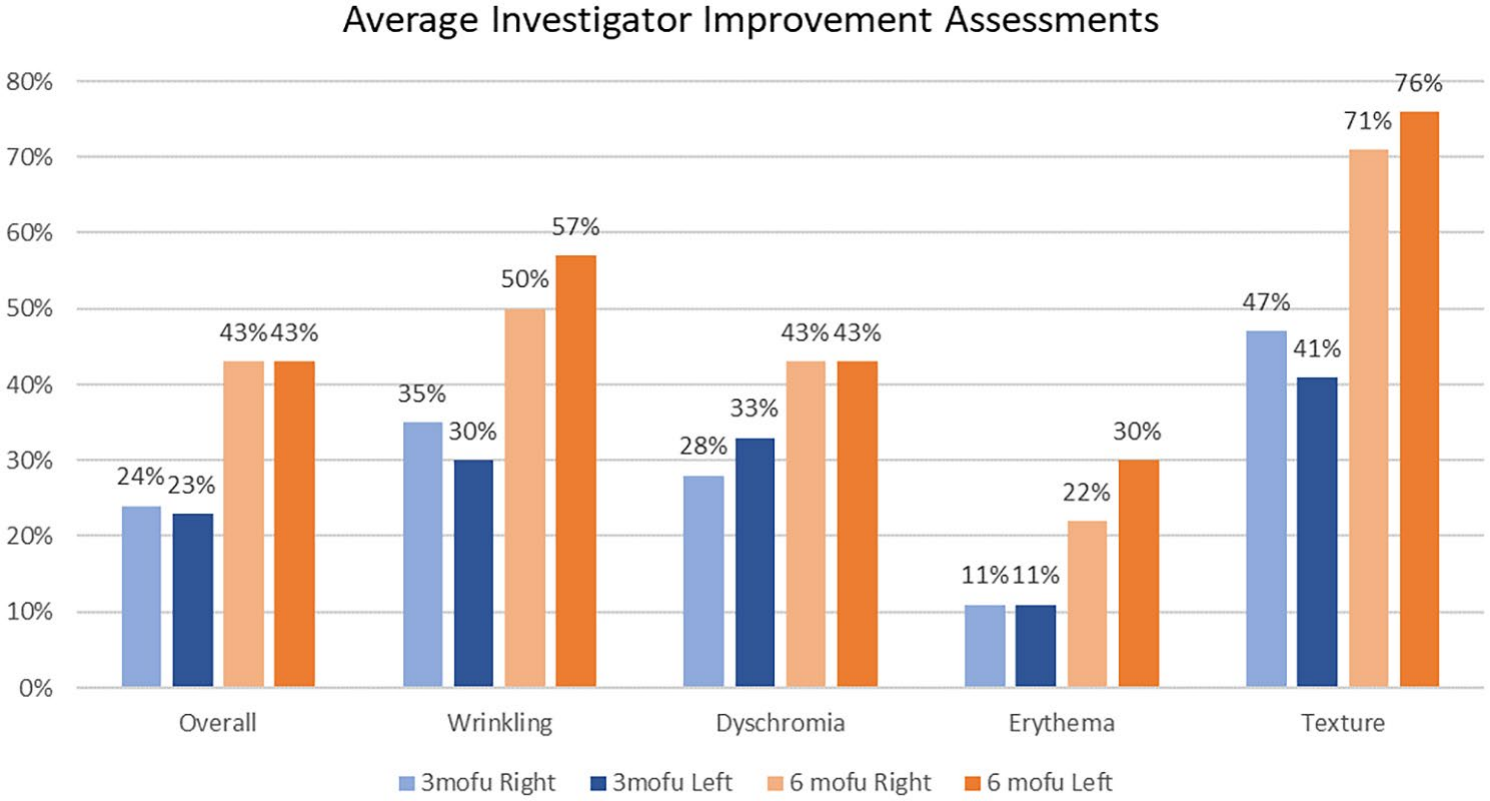
Investigator skin quality assessments, displayed as percent change of scoring compared to baseline scores.

Regarding 3 and 6‐month investigator improvement assessments, all participants achieved improved overall skin appearance, wrinkling, dyschromia, erythema, and texture for both treatment arms. For both PRP and exosomes, the baseline overall average score was 2.12. The post‐treatment overall average PRP scores were 2.70 and 3.04, and exosome scores were 2.68 and 3.14 at 3 and 6 month follow‐up visits, respectively. Overall skin appearance and dyschromia were not significantly different between the PRP and exosome treatment arms at 6 months. However, the exosome treated side showed slightly greater improvement in wrinkling, erythema, and texture (see Figure 1).

**FIGURE 2:**
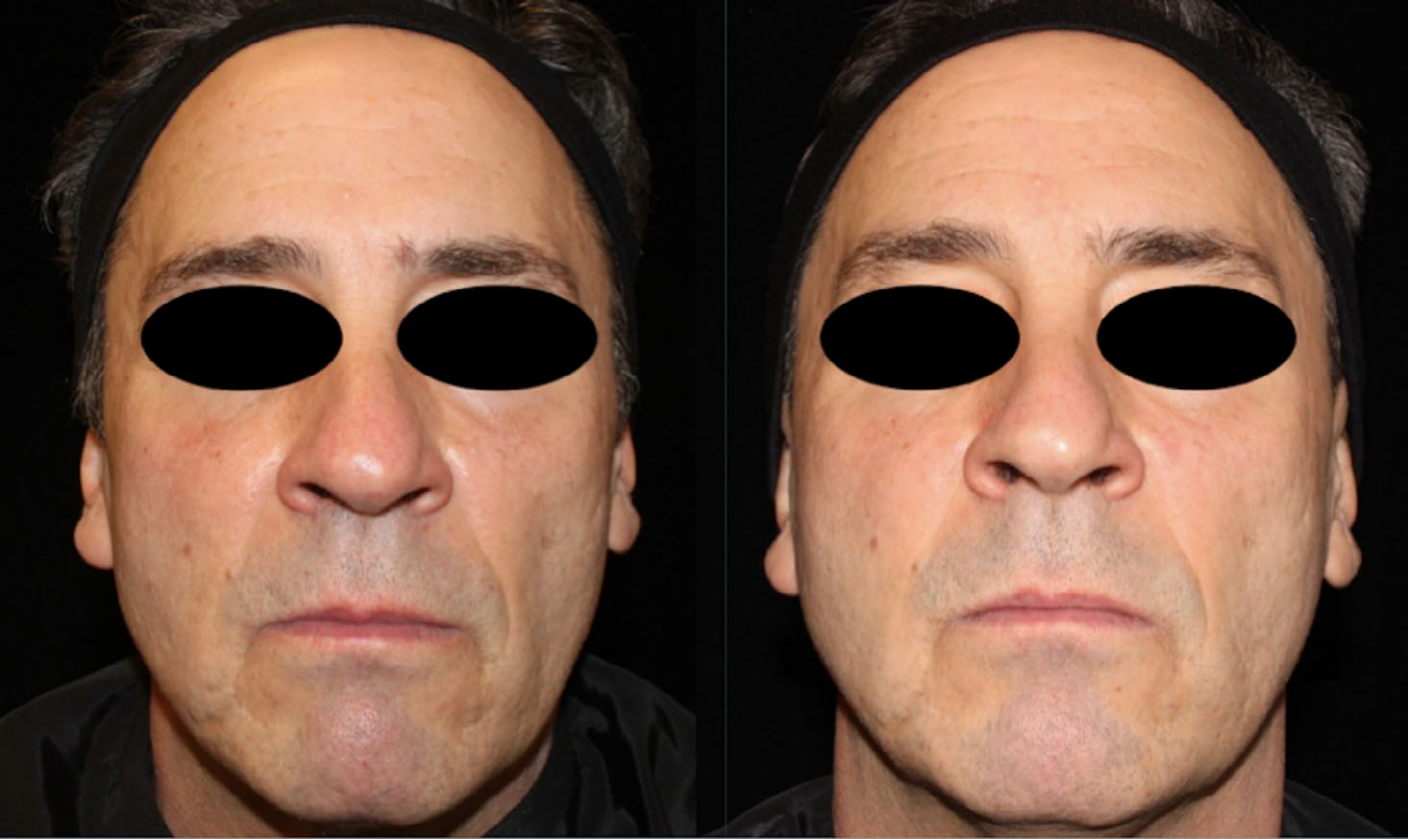
Patient 1 before and after RFMN treatment with exosomes applied to the left side and PRP applied to the right side.

**FIGURE 3:**
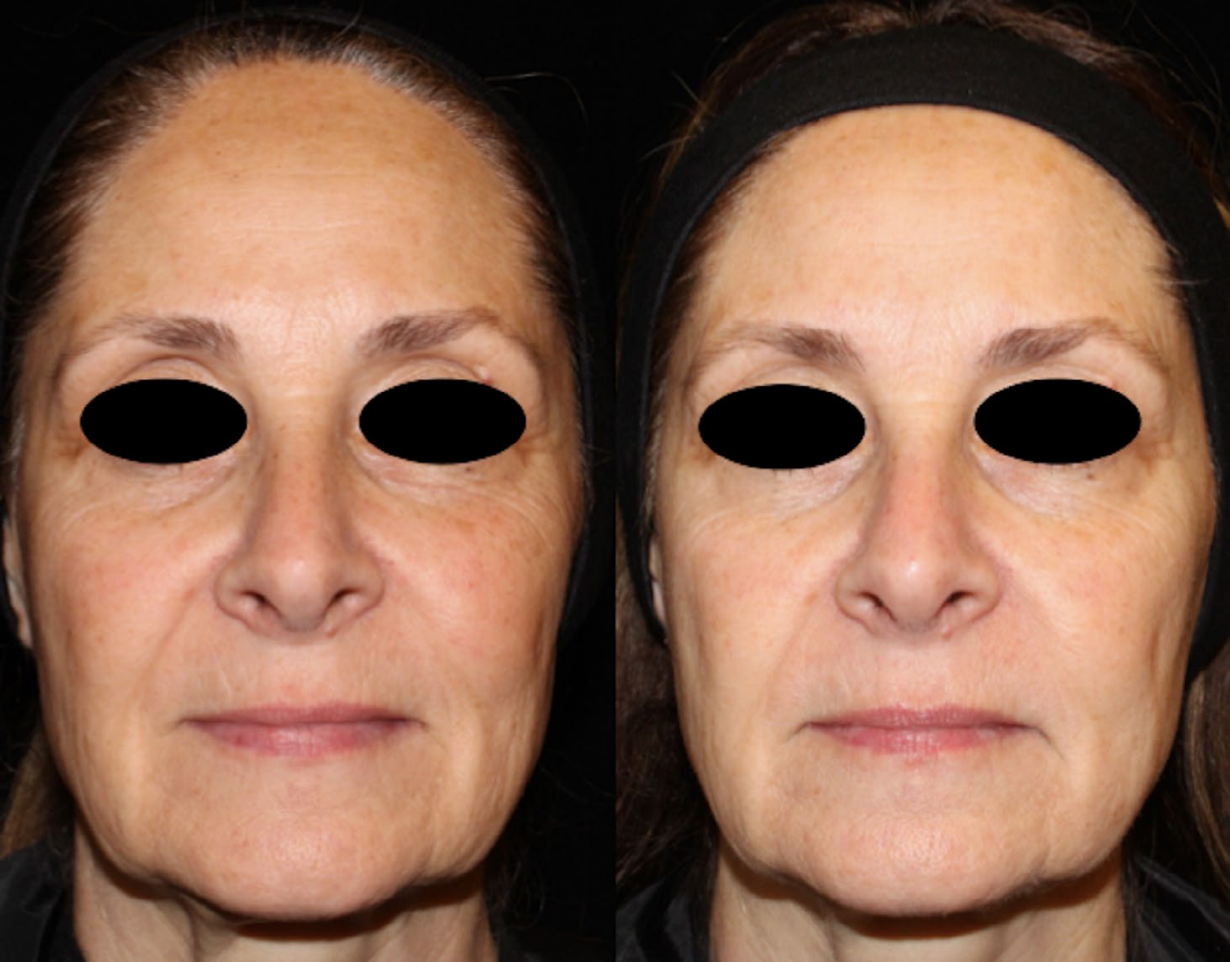
Patient 2 before and after treatment.

**FIGURE 4:**
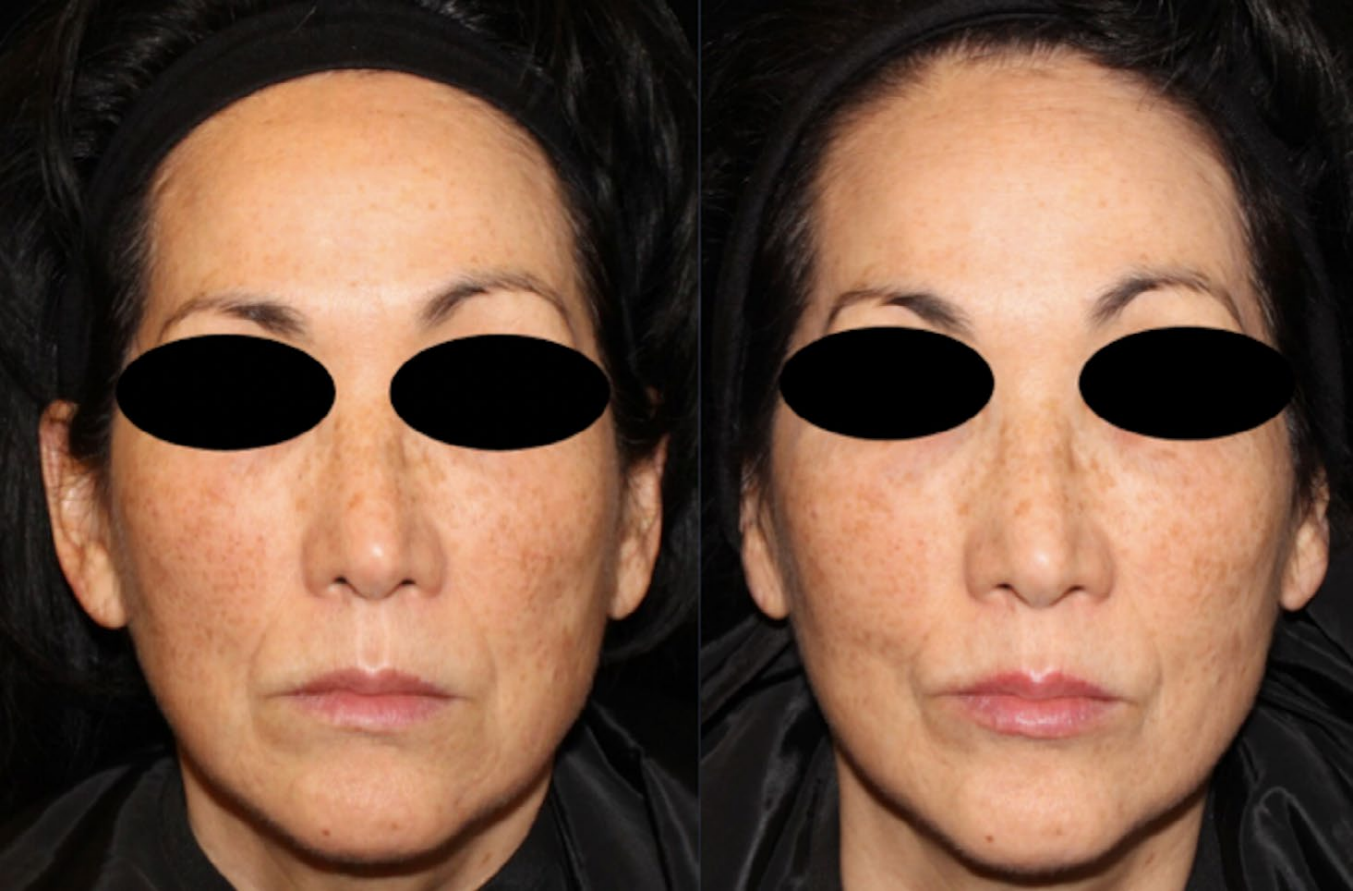
Patient 3 before and after treatment.

**FIGURE 5:**
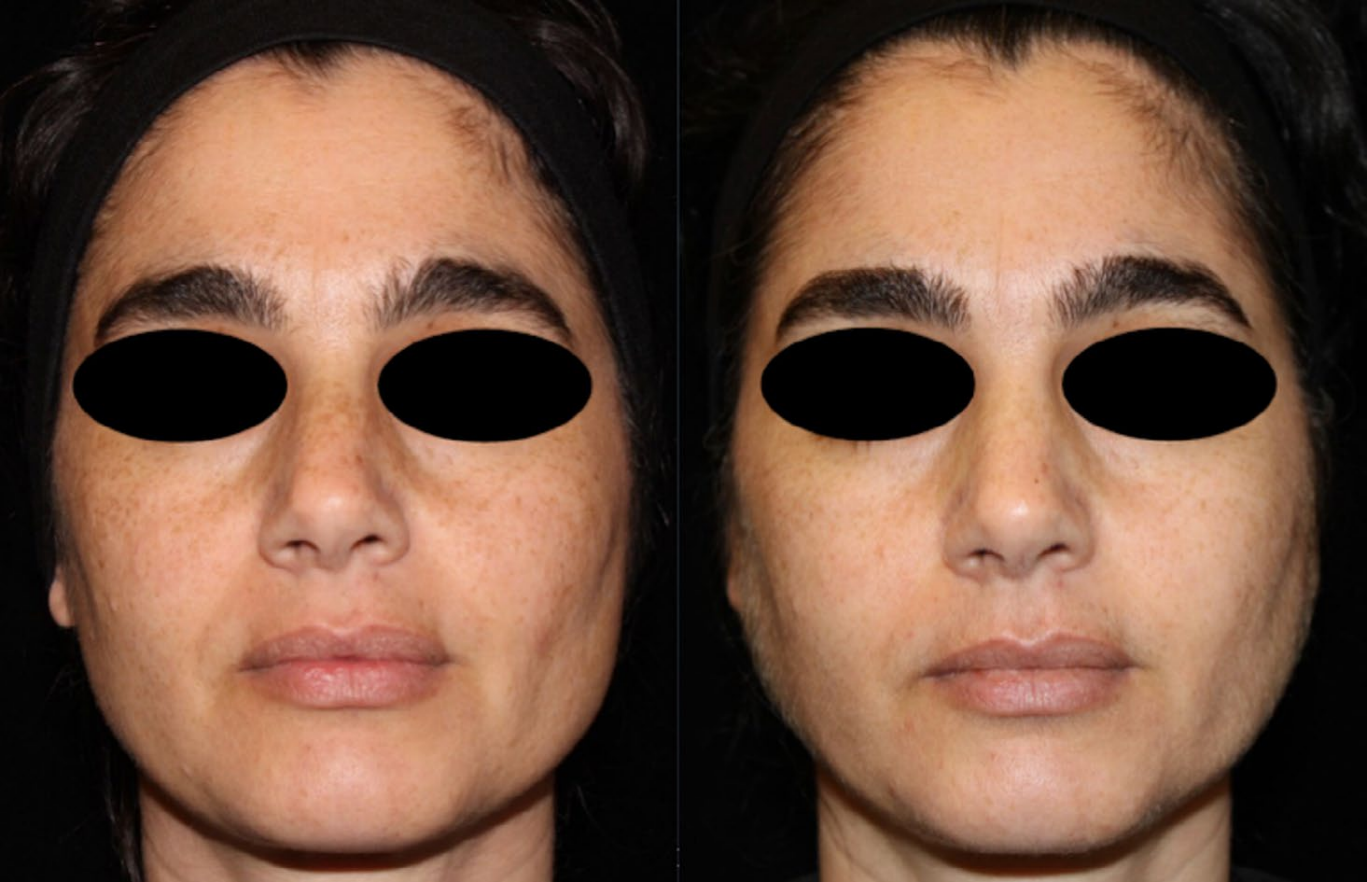
Patient 4 before and after treatment.

Comparing changes in the Griffiths Photonumeric Photoaging Scale, both treatment arms showed a 37% improvement (decrease in score) at 3 month follow up and 22% at 6 month follow up compared to baseline (see Table 1 for Griffiths Photonumeric Photoaging Scale Assessment) (see Figures 2, 3, 4, 5).

**TABLE 1:** Investigator Griffiths' photonumeric photoaging scale assessment.

Average Griffiths' photonumeric photoaging scale scores					
Baseline		3 month follow up		6 month follow up	
Right	Left	Right	Left	Right	Left
4.5	4.5	3.1	3.1	3.6	3.6
Change from baseline		37%	37%	22%	22%

**FIGURE 6:**
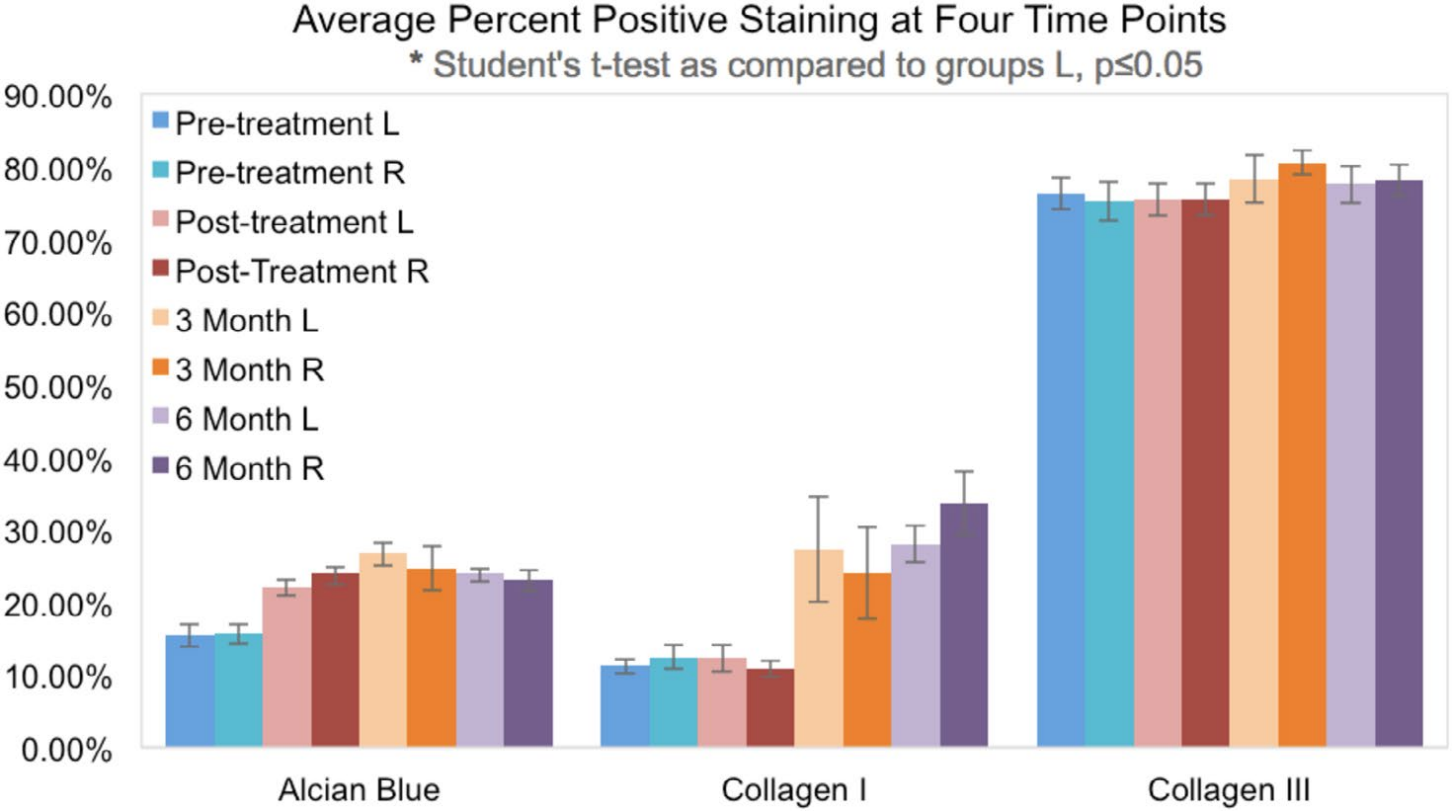
Histological comparison data. L = left side of face, R = right side of face.

**FIGURE 7:**
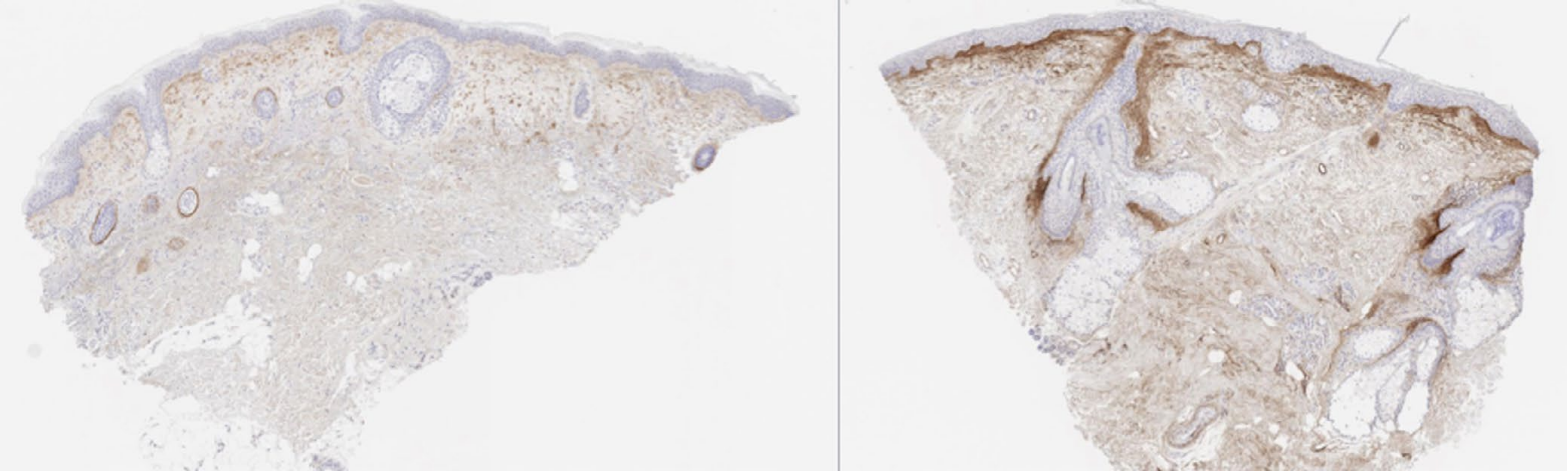
H&E 20×, collagen I staining before and 6 months after exosome treatment.

**FIGURE 8:**
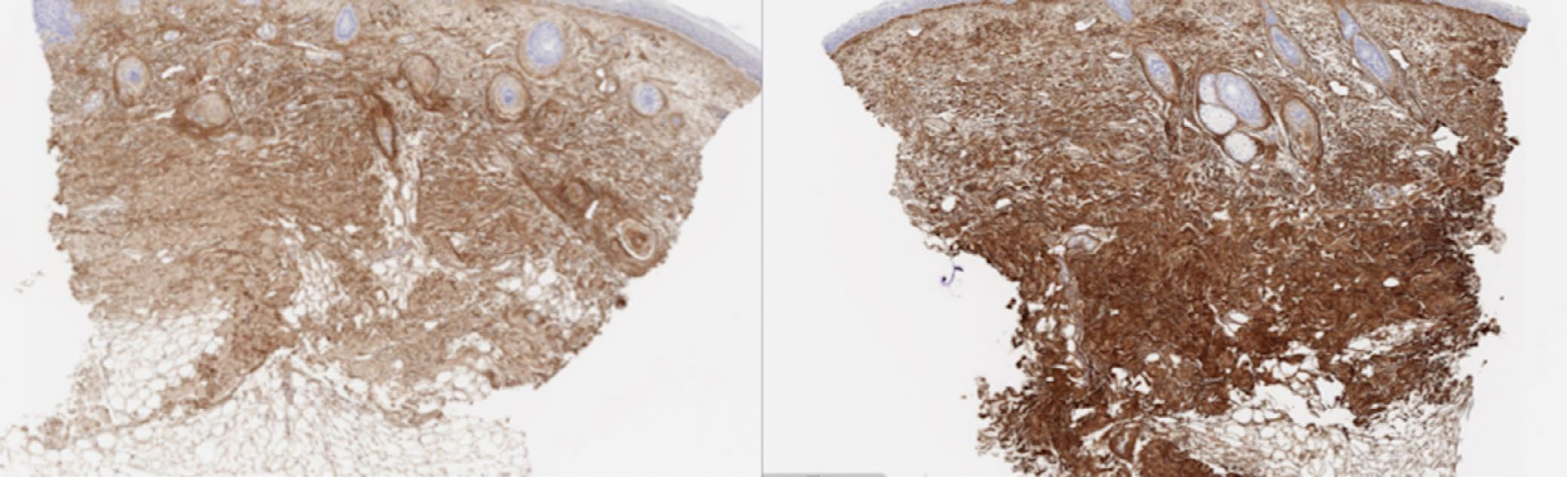
H&E 20×, collagen III histology staining before and 6 months after exosome treatment.

**FIGURE 9:**
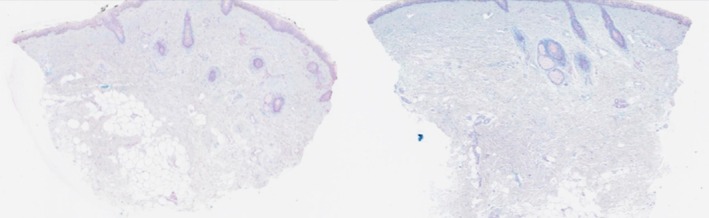
H&E 20×, Alcian blue stain showing GAGs before and 6 months after exosome treatment.

### 4.2 Histological Analysis

A total of 74 skin samples from 10 participants were analyzed. In both the PRP and exosome treatment arms, there was a significant increase in collagen I and glycosaminoglycans when comparing pre‐treatment, 3‐month, and 6‐month post‐treatment slides. Collagen III did not differ in total average percent positive tissue area but did show an increase in the amount of strong positive tissue staining area over time. There were no statistically significant differences between treatment arms (see Figure 6).

For imaging analysis, paired *t*‐tests of equal variance were run for the 9 participants, comparing percent positive pixel counts between the four time points. Alcian Blue significantly increased compared to the pre‐treatment, but not between the post‐treatment groups. Collagen type I percent positive tissue increased in the 3‐month and 6‐month treatments without significant difference between treatment arms. Collagen type III percent positive tissue did not have any significant differences in total tissue. However, there were significant differences in the assigned strength (weak, moderate, or strong) of the positive tissue in the 3‐month and 6‐month treatment compared to the pre‐treatment as there was less weak and moderate tissue and more strong positive tissue (see Figures 7, 8, 9).

## 5 Discussion

Exosomes are extracellular vesicles containing RNA, proteins, amino acids, and lipids. In aesthetic treatments, exosomes exert effects through modulation of inflammation, tissue healing, and fibroblast function [13]. It is important to note that there is high variability in exosome structure and composition, with tissue source and collection method directly impacting their function and efficacy [14].

The product used in the current study contains purified ASC exosomes, which are easily accessible for harvesting, highly stable, and withstand storage [15]. Pre‐clinical data of ASC exosomes have revealed several mechanisms through which skin quality is improved. In UVB‐irradiated human dermal fibroblasts, ASC exosomes decreased matrix metalloproteinases, thereby preventing breakdown of collagen and elastin. Cell proliferation and migration were enhanced while reactive oxygen species and DNA damage were decreased [16, 17].

Of the first published clinical trials, Kwon et al. completed a 12‐week double‐blinded, split‐face study exploring the use of topical ASC exosomes versus a control gel after fractional carbon dioxide laser treatment for acne scarring. The authors found significantly decreased erythema and downtime and noted greater improvement of atrophic acne scars and skin texture on the exosome treated side [18]. Similar to fractional ablative carbon dioxide laser, the RFMN treatment used in our study creates controlled zones of thermal injury resulting in micro‐channels through which topical treatments can penetrate the skin. Our results were congruent, with improved erythema and skin texture after topical ASC exosome treatment.

Recent studies employing the same topical ASC product used in our study have found beneficial effects on facial skin as well. As a post‐microneedling treatment, the exosome product resulted in significantly improved GAIS scores, wrinkling, elasticity, hydration, and pigmentation, with skin biopsies confirming a greater density of collagen and elastic fibers and neocollagenesis than the control arm [9]. For dupilumab‐associated facial erythema, the exosome product reduced erythema, decreased inflammatory markers in the stratum corneum, and increased vascular endothelial growth factor expression [19].

PRP is a widely utilized primary and adjunct treatment for a variety of dermatologic conditions, with alopecia and skin rejuvenation being two common uses. Head‐to‐head trials of PRP and exosomes are lacking in published literature. Hassan et al. found that 1 treatment of human‐derived exosomes (tissue source unspecified) was superior to 5–6 sessions of PRP in stimulating hair growth, with maintenance of results for up to 28 months [20]. To our knowledge, the current study is the first comparative study of exosomes versus PRP for improvement in skin quality and photoaging. Exosome and PRP treatment equally ameliorated key factors of aging skin including wrinkling, dyschromia, erythema, and texture with enhancement of overall skin appearance. Compared to baseline, improvement was maintained at 6 months post‐treatment. Histological analysis demonstrated increased collagen I and GAGs in both treatment arms, confirming the clinical impression. Although the total collagen III staining was unchanged compared to the baseline, the increased percentage of strong positive staining may suggest remodeling of existing collagen III. With aging skin, collagen I and III content and quality decline, with an increasing collagen I/III ratio. The exosome product improved this ratio, which influences skin elasticity and tension [21]. The exosome products were well‐tolerated without any adverse events. Therefore, topical ASC exosomes are a promising PRP alternative as a treatment adjunct.

When considering the patient perspective, exosomes are less invasive as they do not require patient phlebotomy. This may be of particular interest to needle‐averse patients and those who experience vasovagal reactions. Additionally, the exosome product is immediately ready for use and can hasten the office visit duration, which is beneficial for both patients and providers (see Table 2 for PRP/Exosomes Comparison). Post‐procedural skin care is essential to protect the compromised skin barrier and prevent complications, such as infection, irritation, and hyperpigmentation. The post‐treatment product used in this study demonstrated tolerability on compromised skin in the immediate post‐treatment period and can be used as additional exosome delivery therapy.

**TABLE 2:** PRP/exosomes comparison.

Product comparisons		
Treatment	PRP/PRFM	ASC exosomes
Preparation time	15–20 min	< 5 min
Patient discomfort	Mild to moderate	None
Storage requirements	None	Refrigeration until use
Cost per product (specific to this study)	$85 (kit)	$85 (vial)

When considering the source of bioactive substrates, PRP is harvested from the patient's own blood, reflecting their biological age and may be affected by medications and medical conditions causing altered platelet function or thrombocytopenia. Additionally, unstandardized preparation methods may affect PRP's efficacy, resulting in variability in platelet content [4]. Conversely, the exosomes in our study are harvested from healthy donors age 18 to 25 years therefore potentially offering more robust growth factors and regenerative properties. This could account for the slightly greater improvement in wrinkling, erythema, and texture that was observed in the exosome‐treated side.

Of note, our study used a PRP product generated via a platelet‐rich fibrin matrix (PRFM) system, which is a second‐generation autologous concentrate that may offer additional regenerative benefits. The resulting product has a higher concentration of growth factors encapsulated in a fibrin matrix that provides a slower, sustained release of these growth factors. Studies have demonstrated that PRFM may improve clinical outcomes over standard PRP [22].

Regarding safety, there have been several reports of adverse events, all of which developed after injection of exosomes. These reactions include delayed‐onset foreign body and necrotizing granulomas, vascular compromise, skin necrosis, anaphylaxis, and severe infections [23, 24, 25, 26]. Topical application of exosomes, especially following fractional or microneedling procedures, has demonstrated a favorable safety profile in early clinical studies and bypasses the risks associated with needle‐related complications [9]. Currently, the FDA has not cleared any injectable exosome therapy; thus, utilization is limited to topical application.

## 6 Conclusion

Use of exosomes represents a new area of aesthetic dermatology. Exosomes may produce as good or better results than PRP application for skin rejuvenation and without the need for phlebotomy. Limitations of our study include a small sample size, lack of clinical statistical analysis due to the small sample size, and non‐randomization of treatment sides. Larger clinical trials including individuals from a variety of ages and health statuses are needed to confirm the findings of our study, considering the variability of skin healing and collagen synthesis.

## Ethics Statement

The study was approved on February 2, 2023 by the Allendale Institutional Review Board, Division of Regulatory and Technical Associates (Old Lyme, CT 06371). Patients consented to use of clinical photos for this manuscript.

## Conflicts of Interest

The authors declare no conflicts of interest.

## Data Availability

The data that support the findings of this study are available on request from the corresponding author. The data are not publicly available due to privacy or ethical restrictions.
